# Two patients with small chromosome 22q11.21 alterations and central nervous system abnormalities

**DOI:** 10.1186/s13039-015-0200-1

**Published:** 2015-12-30

**Authors:** Carrie Guy, Xianfu Wang, Xianglan Lu, Jin Lu, Shibo Li

**Affiliations:** University of Oklahoma Health Sciences Center, 1122 NE 13 Street, Ste 1400, Oklahoma City, OK 73104 USA

**Keywords:** Semilobar holoprosencephaly, Chiari I malformation, Central nervous system abnormalities, Microarray, Array CGH, Chromosome 22q11, Deletion, Duplication

## Abstract

**Background:**

Central nervous system features have been rarely described to be associated with the small deletion or duplication of chromosome 22q11.21.

**Case presentation:**

We report two patients with chromosome 22q11.21 alterations and central nervous system abnormalities. Features described include semilobar holoprosencephaly in the patient with the small deletion and Chiari I malformation in the patient with the small duplication.

**Conclusions:**

This report will aid in the characterization of the clinical significance of interstitial duplications and deletions on the long-arm of chromosome 22. Areas of future research would benefit from additional analysis of the described regions with inclusion of the phenotypic findings described in this case report to provide additional insight into the pathogenicity of the described alterations.

## Background

We present two patients with similar, small alterations of chromosome 22q11.21, deleted in the first and duplicated in the second patient. Both patients have central nervous system abnormalities rarely reported as features of chromosome 22q11 deletion or duplication syndromes. Deletions and duplications in this region have been previously reported, and with increasing detection with advancing microarray technology, more specific genotype-phenotype associations have been described. Information presented in this report will aid in the characterization of the clinical significance of small interstitial duplications and deletions on the long-arm of chromosome 22.

Deletion of chromosome 22q11 is one of the most widely recognized chromosomal microdeletions. With an incidence of approximately 1/4000 live births, it is the most common, known microdeletion [1]. It is estimated that 5–10 % of cases are inherited, with the remainder being *de novo*. The 22q11 deletion syndrome is associated with a spectrum of features, including congenital heart defects, palatal abnormalities, characteristic facial features, learning difficulties, and immune deficiency [2]. It has also been associated with an increased risk for major psychiatric disorders, particularly schizophrenia [3, 4].

### Central nervous system malformations

Central nervous system malformations, such as holoprosencephaly, are rarely reported with 22q11 deletion syndrome. Often only associated features, such as single median incisor, have been reported in previous case reports. Solitary median central incisor may be an isolated finding, but has also been associated with holoprosencephaly [5]. In 1997, Hall et al. reported a series of 21 cases involving solitary median central incisor, one case of which was later diagnosed with 22q11 deletion syndrome [6]. In 2005, Yang et al. reported a patient with DiGeorge syndrome who was found to have a solitary median central incisor [7]. In a national retrospective study of feto-pathological examinations following a molecular diagnosis of 22q11 deletion, Noel et al. found 10 out of 64 cases had neurological defects, including five with arhinencephalies, another term for holoprosencephaly [8]. Thus, reports of documented holoprosencephaly in patients with 22q11 deletion syndrome have been rarely reported.

Duplication of chromosome 22q11 is also characterized by highly variable features, which may range from normal to mild and may include intellectual disability or learning disabilities, growth retardation, hypotonia, and delayed psychomotor development [9]. Parental testing in prior studies has revealed a high proportion of familial duplications with highly variable parental features [10]. While population data is extremely limited, studies have estimated that duplications are approximately half as common as deletions [10]. However, it is unknown if this is due to the variability of features resulting in ascertainment bias. While Chiari I malformation has been previously reported in 22q11 deletion syndrome [11], this is the first report, to our knowledge, of a patient with 22q11 duplication and Chiari I malformation.

### Molecular genetics of 22q11.21

The frequent and homogeneous deletions and duplications involving chromosome 22q11 has been attributed to the high number of low copy repeats (LCR) in the region, which results in frequent meiotic homologous recombination events [12]. Most patients with 22q11 deletions or duplications have a common 3 megabase (Mb) deletion or a smaller, distal nested 1.5 Mb deletion [13]. The 3 Mb deletion is found in approximately 90 % of individuals with 22q11 deletion syndrome and contains approximately 40 genes, including the gene *TBX1* which has been associated with several main features of 22q11 syndrome including characteristic facial features, cardiac defects, thymic hypoplasia, velopharyngeal insufficiency with cleft palate, and parathyroid dysfunction with hypocalcaemia [2, 13–15]. The 1.5 Mb “nested” deletion is located within the 3 Mb deleted region and includes at least 28 genes including *TBX1* [3, 14, 16]. Recent studies have suggested a higher proportion of maternal origin of *de novo* deletions [17]. Microduplications in this region most commonly include the same 3 Mb region commonly found to be deleted in 22q11 deletion syndrome, though duplication of the smaller, nested 1.5 Mb region and distal duplications have also been reported [10].

Testing for 22q11 deletion syndrome has traditionally been accomplished by fluorescence *in situ* hybridization (FISH) analysis, commonly using the TUPLE1 probe (Vysis/Abbott Laboratories). While traditional FISH analysis will detect the above described small, nested 1.5 Mb deletion, it will not detect other small deletions in this region such as those described below. Thus, microarray analysis has become more commonly utilized when 22q11 deletion syndrome is suspected. With the increase in utilization of microarray, there has been an increase in reports of small deletions not previously detected by FISH analysis.

Out of approximately 7000 samples referred for a variety of indications, the Baylor Cytogenetics laboratory identified 59 individuals with either gains or losses in the 22q11.2 region. Of the 40 individuals with a detected deletion, 33 were the common 3 Mb microdeletion, one was the 1.5 Mb microdeletion, and five had atypical deletions. Of the 19 individuals with duplication, 10 had the 3 Mb microduplication and 9 had smaller duplications. Of the smaller microduplications, two were identified as the common, smaller, nested 1.5 Mb duplication and 7 were unique, including a 1 Mb “nested” duplication from LCR22-3a through LCR22-4, a 1 Mb distal microduplication from LCR22-4 through LCR 22-5, and a 2 Mb distal microduplication from LCR22-4-LCR22-6 [10]. Clinical significance of small duplications of 22q11 remains unclear, as there are few reports of duplications of this area of chromosome 22.

## Case presentation

### Case 1: small duplication 22q11.21

An order was received for microarray based, comparative genomic hybridization (array CGH) on a 3 year-old female due to a delay in social and language development and question of features of autism spectrum disorder. The patient received regular speech therapy due to speech delay (reported to not speak) and occupational therapy. Review of medical records revealed that a hearing evaluation had not been performed due to lack of cooperation. Her diagnoses included attention deficit-hyperactivity disorder, autism spectrum disorder and sensory processing disorder, and developmental language disorder. The patient’s gross motor skills were within the normal range. Normal vision was reported by the parents, but no formal evaluation had been performed. Normal muscle tone and growth was reported by the pediatrician. Orthopedic evaluation revealed no orthopedic concerns. Genetics evaluation revealed no dysmorphic features. The patient was reported to have a latex allergy. Echocardiogram was performed with a normal result. Magnetic resonance imaging (MRI) of the brain revealed Chiari I malformation resulting in moderate effacement of the cerebrospinal fluid space at the cervicomedullary junction. Findings revealed that the cerebellar tonsils project approximately 9 mm into the foramen magnum. See Table 1 for a comparison of the patient’s features to features of 22q11 duplication syndrome described previously.

**Table 1:** Features seen in Case 1 compared to 22q11 duplication syndrome and in Case 2 compared to 22q11 deletion syndrome

Feature	22q11 duplication syndrome	Case 1	22q11 deletion syndrome	Case 2
Craniofacial abnormalities	x	-	+	microcephalic, plageocephalic, overriding sutures, hypotelorism, and depressed nasal bridge
Congenital heart defect	x	-	conotruncal malformations	small patent ductus arteriosus
Palatal defect		NR	+	cleft lip, absent nasal septum, and cleft maxilla papilla
Ophthalmologic abnormalities		-	+	NR
Hearing loss	x	-	+	NR
Urogenital	x	NR	+	grade two hydronephrosis of the right kidney
Intellectual disability/learning disability	x	NR	+	NR
Psychiatric disorder	anxiety	NR	anxiety	NR
Attention deficit hyperactivity disorder		+	+	NR
Autism spectrum disorder		+	+	NR
Behavioral problems	x	NR	+	NR
Speech delay	x	+	+	NR
Growth delay	x	NR	+	NR
Hypotonia	x	NR	+	truncal and appendicular hypotonia
Seizures		NR	+	+
Central nervous system malformation		Chiari I malformation		
Immune deficiency			+	NR

*NR* not reported, + feature present, − feature absent

The patient had one full brother who was 4 years-old and was reported to be healthy with no developmental concerns. Her mother was reported to have had a patent ductus arteriosus (PDA) and patent foramen ovale (PFO) not requiring surgical repair and a history of migraines. She was currently 23 years of age with no major health or developmental concerns. Maternal family history was significant for a cousin with a 5 year-old daughter with autism. Maternal ethnic background was Caucasian (German and Irish) and Native American (Comanche). The father was 25 years of age and reported to be healthy with normal development. There was no known paternal history of developmental delays, autism, or congenital anomalies. Paternal ethnic background was Portuguese and Hawaiian. Consanguinity was denied.

#### Conventional and molecular cytogenetics

**Fig. 1:**
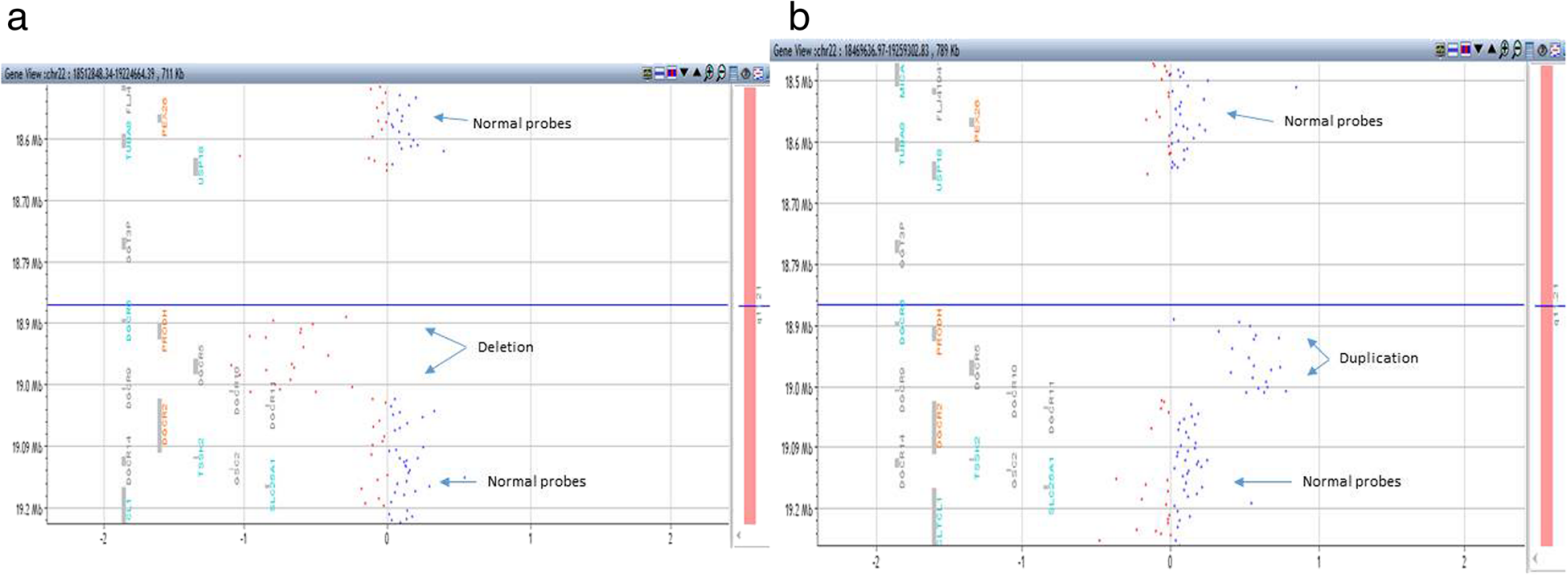
Duplication and deletion on 22q11 in presented cases. **a** 22q11.21 deletion [arr 22q11.21 (18,894,835-19,010,562)x1]. **b** 22q11.21 duplication [arr 22q11.21 (18,894,835-19,010,562)x3]

Array CGH revealed an approximately 115 kb duplication of chromosome 22q11.21 [arr 22q11.21 (18,894,835–19,010,562) × 3] (Fig. 1). Parental analysis was not performed at the time of this report because the family declined further testing. No additional family members had been tested at the time of this report.

A review of DECIPHER (DECIPHER, https://decipher.sanger.ac.uk/) on 5/19/2015 revealed a reported case (285769) of a similar duplication (22:18894835-19010508). However, a second sequence variant (1:187074685-248262713) is also reported and is classified as definitely pathogenic. Phenotypic features include abnormality of the ear, abnormality of the tricuspid valve, aneurysm, duodenal atresia, global developmental delay, narrow mouth, palpebral fissure narrowing on adduction, and thin lips. Decipher case 285666 was also identified as having a similar duplication (chr22:18889039-19010508). Phenotypic features reported include delayed speech and language development, global developmental delay, hypertelorism, low-set ears, motor delay, narrow mouth, short nose, single transverse palmar crease, spontaneous neonatal pneumothorax, and thick lower lip vermilion. However, this case also included three additional alterations (chr8:141415657-146174033, chr10:134001731-135372492, and chr22:16053473-17297403).

Comparison of these previously reported cases in Decipher is limited by the presence of additional alterations, which are absent in the presented patient, Case 1. Given the significant phenotypic variability previously reported to be associated with chromosome 22q11 duplication, it is unclear if the described phenotypes in these prior cases are due to the similar 22q duplication or due to the additional alterations present in these prior cases. Thus, Case 1 reported here is unique in the presence of only this small duplication region.

### Case 2: small deletion 22q11.21

An order for array CGH was received on this two-day old infant following a genetics hospital consultation for seizures and dysmorphic features, specifically unilateral midline defects including cleft lip, absent nasal septum, and cleft maxilla papilla. On exam, she was noted to have overriding sutures, hypotelorism, and a depressed nasal bridge. This female infant was born at 36 weeks gestation to a 21 year-old Caucasian (Irish and German) mother who reported a history of childhood seizures, arthritis, and attention deficit hyperactivity disorder, and a 29 year-old Caucasian (German and Irish) father with a history of possible myocardial infarction, congenital clubfoot, and “sandal gap” toes. Prenatal care included normal maternal serum screening, no reported alcohol or drug use, and reported significant for preeclampsia. Birth weight was 4 lbs, 6.9 oz with Apgar scores of 8 at 1 min and 9 at 5 min. Her head circumference was noted to be 29.0 cm (< 3 %) at two days-old. Consanguinity was denied. Father’s family history was significant for a deceased brother at 12 years-old with an unknown type of heart disease. Paternal family history also included a report of the paternal grandfather and cousins with seizure disorders and an uncle with aortic dissection. Maternal history was significant for a maternal great aunt with early death due to a “hole in the heart”.

Cardiac echocardiogram performed at 1 day of age revealed a small patent ductus arteriosus (PDA) reported to be normal for age. Brain magnetic resonance imaging (MRI) without contrast revealed semilobar holoprosencephaly. Renal ultrasound at two days of age revealed grade two hydronephrosis of the right kidney. She was discharged to home hospice and re-admitted at 1 month 18 days of age for evaluation of breakthrough seizures. Neurology consult revealed that she was unable to track or coo, but was able to lift her head when prone. She was also noted to have truncal and appendicular hypotonia. Electroencephalography (EEG) demonstrated diffuse, nonspecific abnormalities with diffuse sharply contoured waves, suggestive of generalized, nonspecific cortical dysfunction. At discharge, she was also noted to have temperature instability. At 5 months of age, she was noted by otolaryngology to have plagiocephaly and upper airway restriction and severe laryngomalacia, which was treated by laser supraglottoplasty. See Table 1 for a comparison of the patient’s features to features of 22q11 deletion syndrome described previously.

#### Conventional and molecular cytogenetics

Array CGH on peripheral blood revealed an approximately 116 kb deletion of chromosome 22q11.21 [arr 22q11.21 (18,894,835–19,010,562) × 1] (Fig. 1). A review of DECIPHER on 5/19/2015 revealed one case (285903) that also included central nervous system abnormalities. Phenotypic findings included aggressive behavior, dysgenesis of corpus callosum, global developmental delay, hypertelorism, joint laxity, motor delay, narrow palate, pes planus, and short stature. In addition, a review of dbVar (http://www.ncbi.nlm.nih.gov/dbvar/) revealed case nsv931660 with a similar deletion (chr22:18916842-19004772) which was classified as pathogenic and phenotypic features included developmental delay and/or other significant developmental or morphological phenotypes.

Parental array CGH analysis was not performed at the time of this report as it was declined by the family. Fluorescence *in situ* hybridization (FISH) analysis on peripheral blood revealed no interstitial deletion or duplication detected using a DNA probe (LSI TUPLE1 from Vysis/Abbott, Inc.) specific for the DiGeorge syndrome region of chromosome 22q11.2. Routine karyotype analysis on peripheral blood revealed a normal karyotype (46, XX).

## Methods

### Microarray analysis

Human reference genomic DNA was obtained through Agilent Technologies (Agilent Technologies, Santa Clara, CA, USA). The patients’ DNA and reference DNA were labeled with either cyanine 3 (Cy-3) or cyanine 5 (Cy-5) following the standard protocol provided by Agilent. Equivalent labeling DNA products were mixed together and were loaded onto Agilent’s 2 × 400 K oligo microarray chip, which is built based on GRCh37/hg19 (Agilent Technologies, Santa Clara, CA, USA). The slide was incubated in a hybridization oven at 67 °C for 40 h. Slides were then washed and scanned by using a NimbleGen MS 200 Microarray Scanner (NimbleGen System Inc, Madison, WI, USA). Image was analyzed using CytoGenomics 2.7 software (Agilent Technologies, Santa Clara, CA, USA).

### FISH analysis

Fluorescence *in situ* hybridization (FISH) analysis was performed following standard clinical laboratory protocol using a DNA probe (LSI TUPLE1 from Vysis/Abbott Laboratories, Inc.) specific for the DiGeorge syndrome region of chromosome 22q11.2.

### Routine karyotype analysis

Routine karyotype analysis was performed following standard clinical laboratory procedure.

## Conclusions

**Fig. 2:**
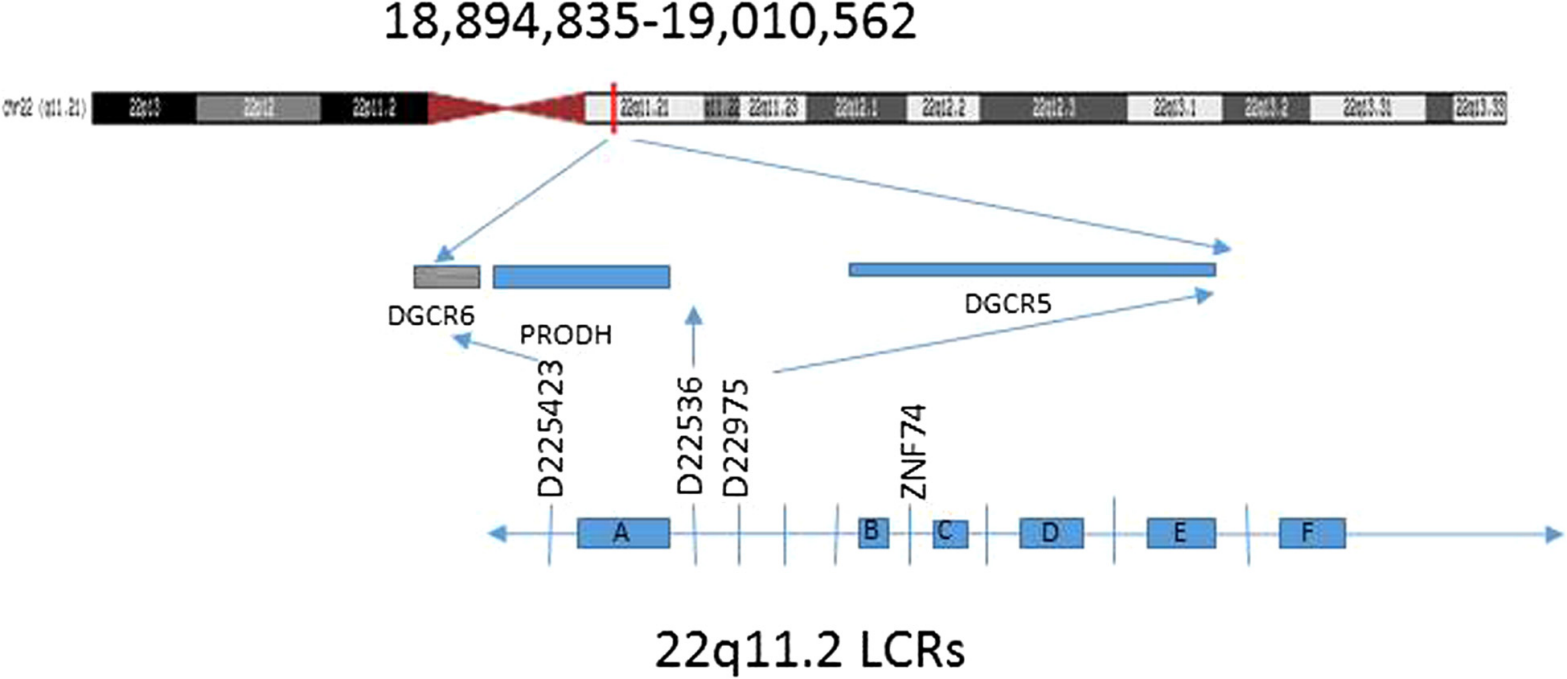
Location of the duplication seen in Case 1 and deletion seen in Case 2. Figure adapted from Shaikh et al. [18] and http://genome.ucsc.edu [19]

We present two cases of 22q11.21 alterations with duplication in one and deletion in the second, and of similar, small size and location (Fig. 2), both with central nervous system abnormalities rarely reported in cases of 22q11 alterations. The first patient has a small (115 kb) duplication of 22q11 [arr 22q11.21 (18,894,835–19,010,562) × 3], attention deficit-hyperactivity disorder, autism spectrum disorder and sensory processing disorder, and developmental language disorder, and was found to have Chiari I malformation on MRI. While Chiari I malformation has been previously reported in 22q11 deletion syndrome [11], this is the first report, to our knowledge, of a patient with 22q11 duplication and Chiari I malformation. In addition, it is the only case identified to have duplication of this region without additional alterations.

The second patient has a small (116 kb) deletion of 22q11 [arr 22q11.21 (18,894,835–19,010,562) × 1], seizure disorder, semilobar holoprosencephaly, microcephaly, cleft lip, grade two hydronephrosis of the right kidney, plagiocephaly, and severe laryngomalacia. While there are previous reports of single median incisor, which is associated with holoprosencephaly, and fetal findings of holoprosencephaly in cases diagnosed with 22q11 deletion syndrome, clinical reports of patients with 22q11 deletion syndrome and holoprosencephaly are rare.

Small deletions and duplications in 22q11 are increasingly being reported with advancing microarray technology. As additional patients are reported, knowledge regarding genotype-phenotype correlations will aid in the clinical management of future patients with these molecular cytogenetic findings. With this report, we aim to contribute to this growing knowledge base and add these reported central nervous system abnormalities to the clinical features described. However, without additional research it is unknown if the above findings are directly related to the described alteration of chromosome 22q11 or coincidental findings, given the phenotypic variability of both deletions and duplication of 22q11 region. Thus, the pathogenicity of the described alterations remains unclear. Areas of future research would benefit from additional analysis of the described regions with inclusion of the phenotypic findings described in this case report.

## Consent

IRB/consent is not required according to the policy of the University of Oklahoma Health Sciences Center, as all patient information is de-identified and samples were received following standard of care clinical evaluation.

## Abbreviations

array CGH: microarray based comparative genomic hybridization
EEG: electroencephalography
FISH: fluorescence *in situ* hybridization
kb: kilobase
LCR: low copy repeats
Mb: megabase
MRI: magnetic resonance imaging
PDA: patent ductus arteriosus
PFO: patent foramen ovale
